# Systematic review and meta-analysis on the effectiveness of ultrasound-guided versus landmark corticosteroid injection in the treatment of shoulder pain: an update

**DOI:** 10.1007/s40477-022-00684-1

**Published:** 2022-05-06

**Authors:** Mohamed Magdy ElMeligie, Nashwa M. Allam, Radwa M. Yehia, Ahmed A. Ashour

**Affiliations:** 1 Present Address: Department of Basic Sciences, Faculty of Physical Therapy, Ahram Canadian University, 4th Industrial Zone, Banks Complex، 6th of October City, Giza, Egypt; 2 Department of Physical Therapy for Orthopedics & Sport Injuries, October 6 University, Central Axis, 6th of October City, Giza, Egypt; 3 Department of Physical Therapy for Women’s Health, Faculty of Physical Therapy, October 6 University, Giza, Egypt; 4 Department of Biomechanics, Faculty of Physical Therapy, October 6 University, Central Axis, 6th of October City, Giza, Egypt

**Keywords:** Shoulder, Ultrasound, Corticosteroid, Landmark, Systematic review, Meta-analysis

## Abstract

**Background:**

Corticosteroid (CS) can be injected in a blind fashion (landmark-guided) or with ultrasound (US) guidance, and this may contribute to varying clinical results. We conducted this systematic review and meta-analysis to assess the effectiveness of US-guided versus landmark CS injections in the treatment of adult patients with shoulder pain.

**Methods:**

We searched MEDLINE (via PubMed), Scopus, Web of Science, EBSCO, and Cochrane Library for randomized controlled trials (RCTs) comparing US-guided versus landmark CS injection regarding visual analogue scale (VAS), functional scores, disability scores, abduction degree, and side effects. The data were pooled as mean difference (MD), standardized mean difference (SMD), or risk ratios (RRs), with 95% confidence intervals (CIs), using R software (meta package 4.9-0) for windows. Subgroup analysis and leave-one-out analysis were conducted.

**Results:**

Eighteen RCTs, with a total of 1010 patients, were included in this meta-analysis. The pooled estimate favored the US-guided over landmark CS injection in terms of the mean change of VAS between 6 weeks and baseline (SMD = − 0.48, 95% CI [− 0.79, − 0.17]), the shoulder functional scores (SMD = 0.35, 95% CI [0.05, 0.65]) and shoulder abduction degree (MD = 8.78, 95% CI [3.11, 14.46]). Whilst no significant difference was found between the compared group regarding the overall shoulder disability scores (SMD = − 0.51, 95% CI (− 1.25, 0.22]) and side effects (RR = 0.45, 95% CI [0.15, 1.34]). None of the eligible study analyzed the cost-effectiveness of the US-guided method compared with the landmark method for CS injection.

**Conclusion:**

Our analysis showed that US-guided CS injection was effective in the treatment of various shoulder diseases. Further research on the cost-effectiveness of US-guided CS methods is needed.

**Supplementary Information:**

The online version contains supplementary material available at 10.1007/s40477-022-00684-1.

## Introduction

Shoulder pain is a common cause of musculoskeletal pain, accounting for the third most common pain in orthopedic practice [1]. Corticosteroid (CS) injections are commonly used in the treatment of shoulder pain regardless of the underlying cause (e.g., impingement syndrome, bursitis, adhesive capsulitis, and rotator cuff disease). It has been reported that CS injection improves functional outcomes and compliance with physical therapy [2].

Shoulder injections are either performed in a blind fashion via anatomical landmarks to guide needle placement or via image guidance, such as ultrasonography (US) [3, 4]. There has been considerable debate regarding the most efficacious method of injection in the treatment of shoulder diseases. The landmark posterolateral approach is commonly preferred. However, the belief that correct needle placement and drug administration leads to better clinical recovery has resulted in increased use of US-guided injections. Previous research reported improvement in shoulder outcomes irrespective of the needle positioning was in the correct structure or not [5]. Others have demonstrated better clinical outcomes with US-guided injections [6, 7]. On the other hand, some studies disproved its superiority to landmark injection [8, 9]

To date, no definite guidance on optimal clinical practice exists, and it is unclear whether US-guided injection improves patient-related outcomes in shoulder pain. The null hypothesis of this study was that there would be no difference in shoulder pain, functional outcome, disability scores, and abduction degree in patients receiving US-guided CS injections compared to landmark injections in the treatment of various shoulder diseases. Therefore, in this systematic review and meta-analysis, we aimed to investigate the effectiveness and safety of US-guided versus landmark CS injections in the treatment of adult patients with shoulder pain.

## Methods

The current systematic review and meta-analysis study was reported according to the Preferred Reporting Items for Systematic Reviews and Meta-Analyses (PRISMA) [10].

### Search strategy

We performed a systematic search of the MEDLINE (via PubMed), Scopus, Web of Science, EBSCO, and Cochrane Library of Clinical Trials databases up to July 3, 2021. The search was executed using the keywords ultrasound, blind, landmark, image-guided, steroid*, corticosteroid*, shoulder*, capsulitis, bursitis, impingement syndrome. We used the Medical Subject Headings terms where applicable. Also, we searched manually the references of included studies for any additional eligible RCTs. Search strategies in different databases are reported in Supplementary file.

### Study selection

The title/abstract and full text of retrieved publications were screened by two investigators independently and in duplicate. Inclusion was restricted to RCTs published in English that randomized adult patients with shoulder pain (aged ≥ 18 years) to US-guided vs landmark CS. We excluded observational studies, case reports, reviews, editorials, commentaries, abstracts, thesis, and conference proceedings.

### Data extraction

For each included RCT, data on study and patient characteristics were extracted independently using a data collection form. Extracted study and patient characteristics included the first author’s name, year of publication, country, study design, number of patients in each group, mean age, female percentage, shoulder disease, shoulder function score, and follow-up duration.

The following outcomes were extracted: changes between baseline and 6 weeks of the visual analogue score (VAS, the scale of 0–10 or 0–100 rating pain, with the higher score, the more severe pain), the Disabilities of the Arm, Shoulder, and Hand (DASH, a scale score ranging from 0, no disability to 100, most severe disability), Shoulder Disability Questionnaire (SDQ, a 16 items score designed to evaluate functional status limitation in patients with shoulder disorders), Shoulder Pain and Disability Index (SPADI, 13 items assessing pain level and extent of difficulty, the pain subscale has 5-items and the Disability subscale has 8-items), Constant-Murley Score (CMS, score ranging from 0 to 100 points, representing worst and best shoulder function), Oxford score (a 12-item patient-reported score), shoulder abduction degree, side effects such as post-injection pain and skin peeling, and cost-effectiveness analysis.

### Risk of bias assessment

The quality of included RCTs was assessed using the Risk of Bias Tool developed by the Cochrane Collaboration [11]. For each RCT was assigned a score of high, low, or unclear to each of the following domains: sequence generation; allocation concealment; blinding of participants, personnel, and outcome assessors; incomplete outcome data; selective outcome reporting; and other potential sources of bias. Disagreements were resolved by consensus.

### Statistical analysis

Pooled data were calculated as standardized mean difference (SMD), mean difference (MD), or risk ratio (RR) with their corresponding 95% confidence interval (CI). Statistical analyses were conducted using R software, version 4.0.3, for Windows. *I*^2^ and *Q* were calculated to assess heterogeneity among the included RCTs. *I*^2^ was estimated via a weighting approach using a Mantel–Haenszel fixed-effects approach. Sensitivity analyses were conducted using Mantel–Haenszel fixed-effects models to assess the effect of using a random-effects model on our estimates. Leave-one-out-meta-analysis was performed to assess the contribution of each study to the overall model. The funnel plot method was used to assess the publication bias among the include studies.

## Results

### Search results

**Fig. 1:**
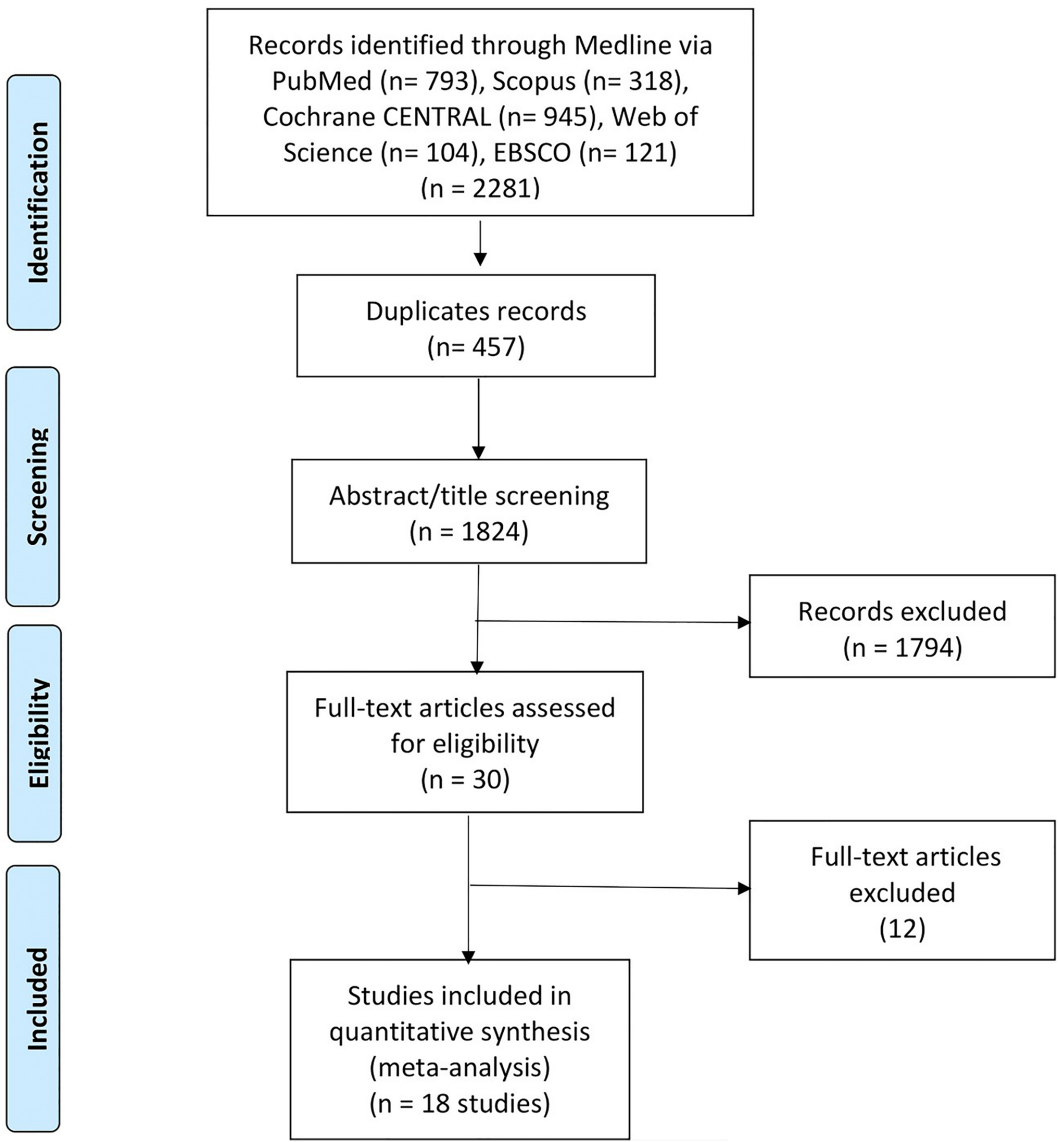
The PRISMA flow diagram of study selection

Our systematic search identified 2281 relevant publications. There were 457 duplicates, leaving 1824 to be screened by title and abstract. We identified 30 publications for full-text review. Of those, 18 met our prespecified criteria for inclusion in our meta-analysis. The PRISMA flow diagram of study selection is shown in Fig. 1. A list of excluded studies with the main reason of exclusion is reported in Supplementary file.

### Study characteristics

Eighteen RCTs [8, 9, 12–27], with a total of 1010 patients who received US-guided CS or landmark CS injection were included in the meta-analysis. Sample sizes ranged from 28 to 100 patients. Follow-up duration ranged from 1 week to 32.5 weeks. Three studies were conducted in Turkey [9, 20, 21], two in South Korea [22, 23], two in Taiwan [24, 25], two in Iran [26, 27], and one each in United Kingdom [12], Spain [13], China [14], Japan [15], Switzerland [16], Australia [8], Ireland [17], Greece [18], and India [19]. The major characteristics of enrolled patients in each RCT are detailed in Table 1.

**Table 1:** Summary table and baseline characteristics of included studies

Author, year	Country	Study design	Population (US-guided/blind CS injection)	Age, mean (US-guided/blind CS injection)	Female % (US-guided/blind CS injection)	Diagnosis	Shoulder function scores	Follow up
Cho et al. (2021) [22]	South Korea	RCT	45/45	–	–	Primary frozen shoulder	ASES	12 weeks
Akbari et al. (2020) [20]	Turkey	RCT	14/14	40.75/42.25	57.1/64.3	Subacromial impingement syndrome	DASH, CMS	4 weeks
Yiannakopoulos et al. (2020) [18]	Greece	RCT	22/22	41.5/43.9	54.5/40.9	Bicipital tendinosis	QuickDASH	6 weeks
Bhayana et al. (2018) [19]	India	RCT	30/30	44.53/42.03	56.6/33.3	Rotator cuff syndrome	CMS	3 months
Raeissadat et al. (2017) [26]	Iran	RCT	20/21	57.8/59.9	35/38.1	Shoulder adhesive capsulitis	Oxford shoulder score	4 weeks
Cole et al. (2015) [8]	Australia	RCT	28/28	46/42	50/64	Subacromial impingement syndrome	ASES	6 weeks
Haghighat et al. (2015) [27]	Iran	RCT	20/20	50.45/52.3	60/65	Subacromial impingement syndrome	SPADI	6 weeks
Saeed et al. (2014) [17]	Ireland	RCT	50/50	57.7	65	Subacromial impingement syndrome	Shoulder function tests	12 weeks
Hsieh et al. (2013) [24]	Taiwan	RCT	46/46	57.59/55.87	58.7/63	Chronic subacromial bursitis	SPADI, SDQ, SF-36	4 weeks
Dogu et al. (2012) [9]	Turkey	RCT	23/23	55.17/56.74	65.2/69.6	Subacromial impingement syndrome	Constant ADLS, SDQ	6 weeks
Zufferey et al. (2012) [16]	Switzerland	RCT	32/33	53/54	40.6/45.5	Bursitis, fluid or synovitis	CMS	6 weeks
Hashiuchi et al. (2011) [15]	Japan	RCT	15/15	59.7/67.8	46.7/66.7	Biceps tendinitis	–	–
Zhang et al. (2011) [14]	China	RCT	53/45	49/43	35.8/35.6	Biceps tendinitis	CMS	32.5 weeks
Panditaratne et al. (2010) [12]	United Kingdom	RCT	41/17	54	62.1	Chronic subacromial bursitis	Oxford shoulder score	8 weeks
Lee et al. (2009) [23]	South Korea	RCT	20/20	53.1/54.1	55/50	Adhesive capsulitis	General shoulder function tests	6 weeks
Ucuncu et al. (2009) [21]	Turkey	RCT	30/30	52.1/52.9	73.3/73.3	Impingement syndrome; subacromial subdeltoid bursitis; rotator cuff lesion	CMS	6 weeks
Chen et al. (2006) [25]	Taiwan	RCT	20/20	53	33.3	Subacromial bursitis	–	1 week
Naredo et al. (2004) [13]	Spain	RCT	21/20	52.9/51.9	71/60	Impingement syndrome, subacromial-subdeltoid bursitis, rotator cuff lesions	Shoulder Function Assessment scale	6 weeks

*US-guided* ultrasound-guided, *CS* corticosteroid, *RCT* randomized controlled trial, *DASH* the Disabilities of the Arm, Shoulder, and Hand, *ASES* the American Shoulder and Elbow Surgeons score, *CMS* Constant-Murley Score, *ADLs* activities of daily living, *SDQ* Shoulder Disability Questionnaire, *SPADI* Shoulder Pain and Disability Index, *SF-36* the 36-item Short-Form Health Survey

### Quality assessment

Overall, studies had a low risk of bias, as assessed by the Cochrane Risk of Bias Tool. Random sequence generation was used in 16 studies. Blinding was unclear in nine studies. Incomplete outcome data and selective outcome reporting were kept unbroken in 17 studies. The summary of the risk of bias assessment is shown in Supplementary file.

### Patient characteristics

patient characteristics were similar across studies, Table 1. Most participants were in their middle 40–60 s. Females represented 36–73% of trial participants. The patients were diagnosed with subacromial impingement syndrome (seven studies), subacromial bursitis (four studies), adhesive capsulitis (two studies), biceps tendonitis (two studies), frozen shoulder (one study), rotator cuff syndrome (one study), and biceps tendinosis (one study).

## Outcomes

### Changes of VAS

**Fig. 2:**
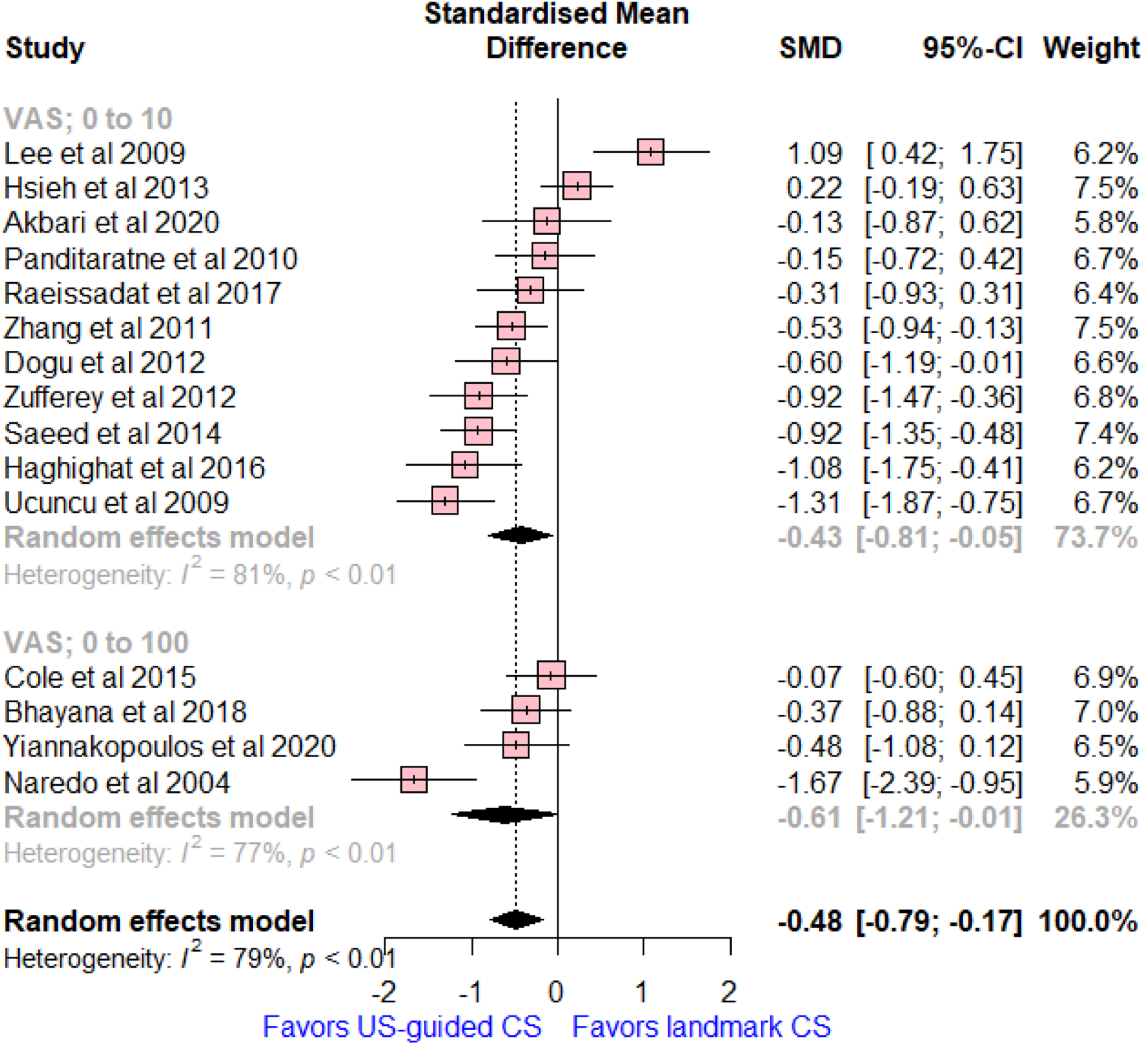
Forest plot comparing US-guided vs landmark CS injection regarding differences of mean changes of VAS score between baseline and 6 weeks

Fifteen studies reported on VAS score, with a total of 850 patients. There was a significant reduction in the mean change of VAS between 6 weeks and baseline in the US-guided CS compared to landmark CS (SMD = -− 0.48, 95% CI [− 0.79, – 0.17]; *I*^2^ = 79%, *p* < 0.01, Fig. 2). Subgroup analysis by VAS score 0–10 or 0–100 was consistent with the overall effect size (For VAS, 0–10, SMD = − 0.43, 95% CI [− 0.81, − 0.05]; *I*^2^ = 81%, *p* < 0.01, and for VAS, 0–100, SMD = − 0.61, 95% CI [− 1.21, − 0.01]; *I*^2^ = 77%, *p* < 0.01).

### Changes of shoulder function score

**Fig. 3:**
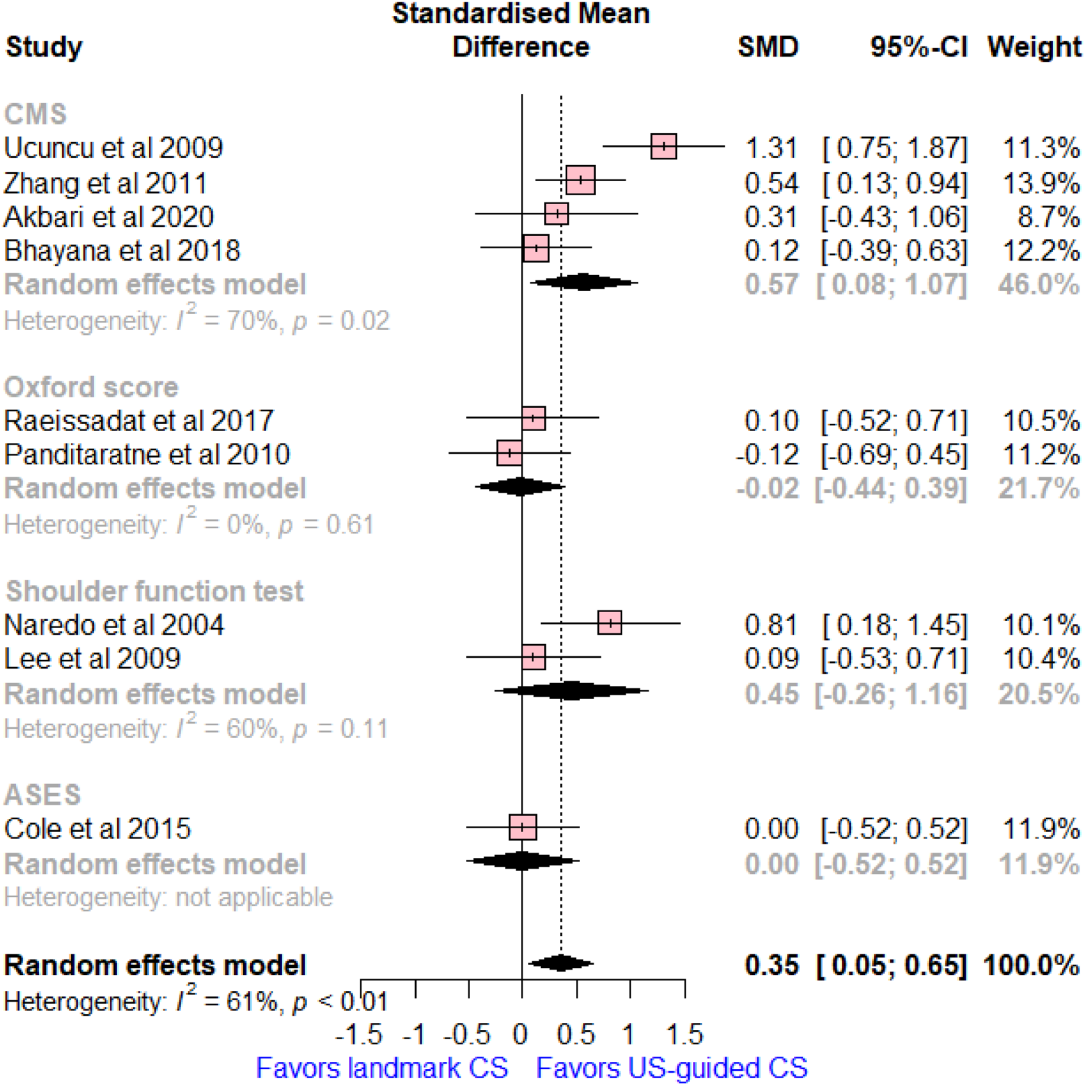
Forest plot comparing US-guided vs landmark CS injection regarding differences of mean changes of shoulder function scores between baseline and 6 weeks

Nine studies reported on various shoulder function scores, with a total of 482 patients. US-guided CS has a significant improvement in mean changes of the shoulder function scores at 6 weeks follow up compared to landmark CS (SMD = 0.35, 95% CI [0.05, 0.65]; *I*^2^ = 61%, *p* < 0.01, Fig. 3). Subgroup analysis by the type of functional score was kept true in the CMS score (SMD = 0.57, 95 CI [0.08, 1.07]; *I*^2^ = 70%, *p* = 0.02), while no significant differences were reported between the compared groups regarding Oxford score (SMD = − 0.02, 95% CI [− 0.44, 0.39]; *I*^2^ = 0%, *p* = 0.61), general shoulder function test (SMD = 0.45, 95% CI [− 0.26, 1.16]; *I*^2^ = 60%, *p* = 0.11), and ASES score (SMD = 0.00, 95% CI [− 0.52, 0.52]).

### Shoulder disability scores

**Fig. 4:**
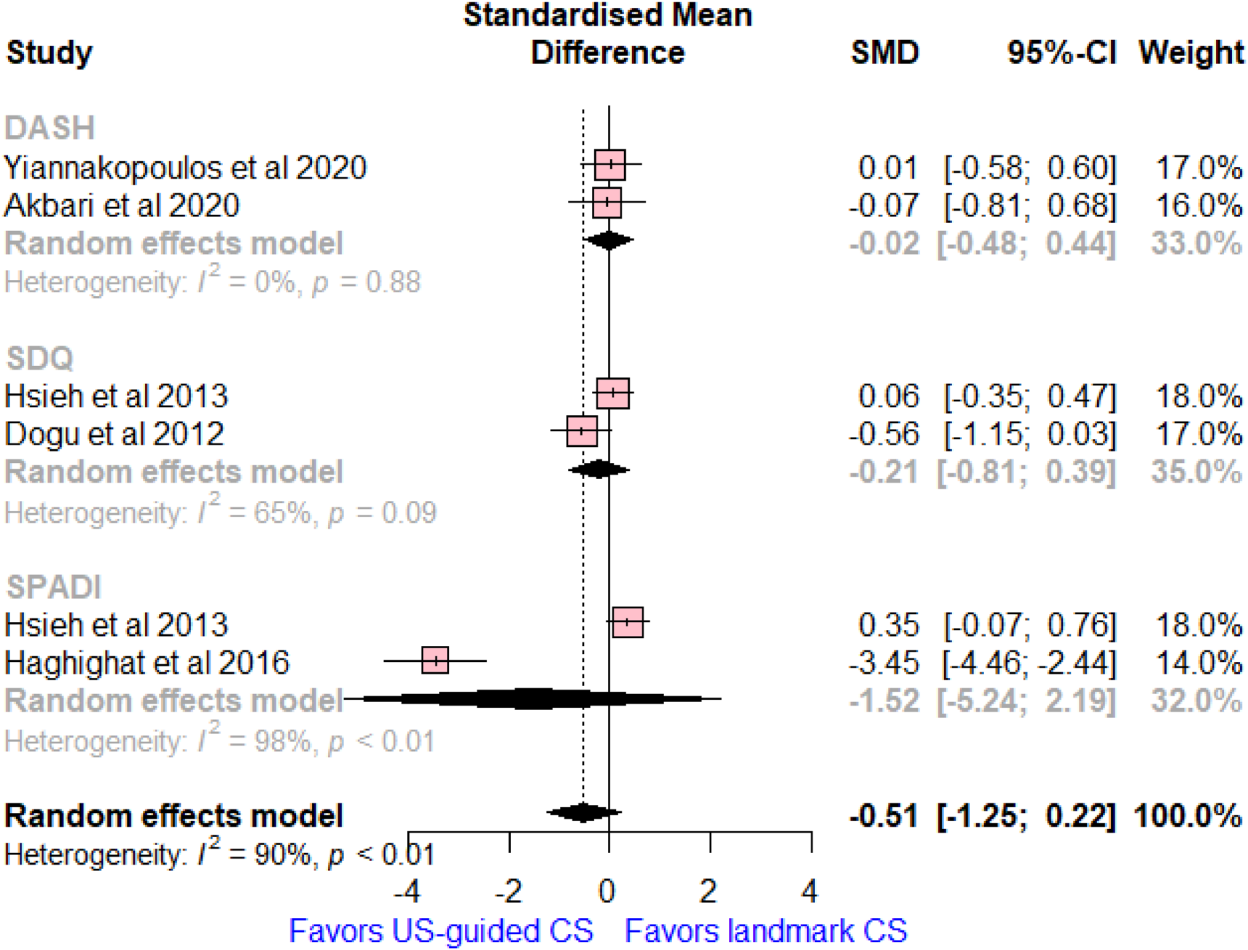
Forest plot comparing US-guided vs landmark CS injection regarding differences of mean changes of shoulder disability scores between baseline and 6 weeks

Six studies reported on shoulder disability scores, with a total of 342 patients. No significant difference was found between the compared group related to the overall shoulder disability scores (SMD = − 0.51, 95% CI (− 1.25, 0.22]; *I*^2^ = 90%, *p* < 0.01, Fig. 4). This effect was consistent with subgroup analysis by the type of disability score in terms of DASH score (SMD = -− 0.02, 95% CI [− 0.48, 0.44]; *I*^2^ = 0%, *p* = 0.88), SDQ score (MD = − 0.21, 95% CI [− 0.81, 0.39]; *I*^2^ = 65%, *p* = 0.09), and SPADI disability subscale (SMD = − 1.52, 95% CI [− 5.24, 2.19]; *I*^2^ = 98%, *p* < 0.01).

### Changes of shoulder abduction degree

**Fig. 5:**
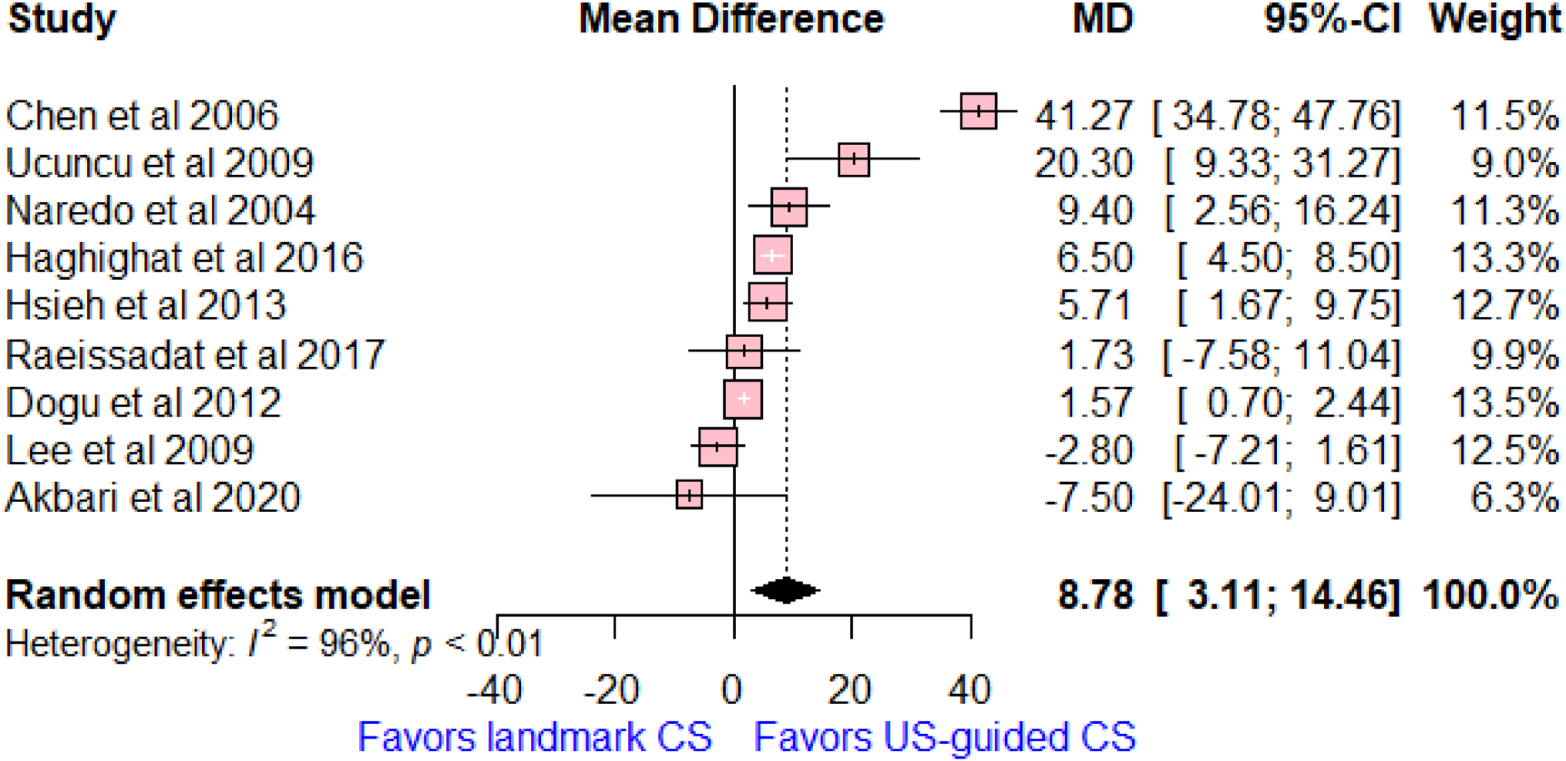
Forest plot comparing US-guided vs landmark CS injection regarding differences of mean changes of shoulder abduction degree between baseline and 6 weeks

Nine studies reported on shoulder abduction degree, with a total of 428 patients. There was a significant increase in the shoulder abduction degree in the US-guided CS group compared to the landmark group (MD = 8.78, 95% CI [3.11, 14.46]; *I*^2^ = 96%, *p* < 0.01, Fig. 5).

### Side effects

**Fig. 6:**
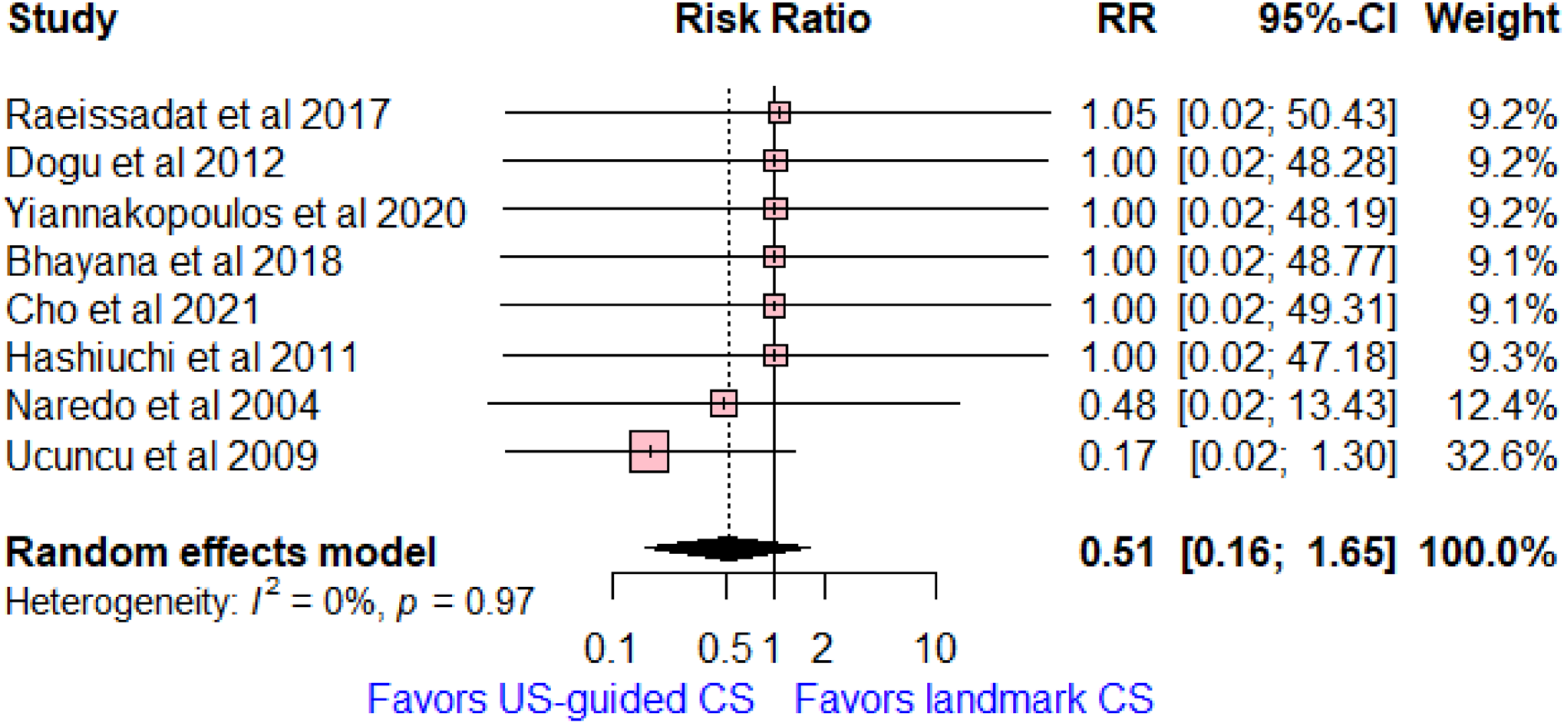
Forest plot comparing US-guided vs landmark CS injection regarding side effects

Eight studies reported side effects (such as skin peeling, vascular injury, or infection), with a total of 412 patients. No significant difference was found between the compared groups in terms of side effects (RR = 0.45, 95% CI [0.15, 1.34]; *I*^2^ = 0%, *p* = 0.97, Fig. 6).

### Cost-effectiveness

None of the eligible studies analyzed the cost-effectiveness of US-guided application of CS injection.

### Leave-one-out-analysis

We observed significant evidence of heterogeneity in most outcomes with *I*^2^ more than 50% and *P* less than 0.1. However, the heterogeneity was not resolved by the conduction of sensitivity analysis or subgroup analysis. Therefore, a leave-one-out meta-analysis was conducted to assess the contribution of each study to the overall model. No study affected the overall estimate in the leave-one-out-study analysis and there was no marked difference in results, suggesting that the results of the current study were not driven by a single study, Supplementary file.

### Publication bias

The funnel plot method was used to assess any evidence of publication bias. No significant publication bias was observed, and funnel plots were symmetrical in all outcomes, Supplementary file.

## Discussion

The findings of this meta-analysis demonstrated that US-guided steroid injection was effective in the treatment of patients with several shoulder diseases. Ultrasound-guided steroid injection was associated with significant pain reduction, shoulder function gain, and shoulder abduction improvement at 6 weeks of follow-up in comparison to landmark steroid injection. However, both US-guided and landmark steroid injections have comparable effects in respect to shoulder disability scores and adverse events.

The positive effect of steroids is probably due to their anti-inflammatory effect. Improper injection of steroids may lead to incomplete response due to further diffusion of steroid away from its target site. Ultrasound is a safe and reliable method that ensures accurate placement of the needle and delivery of the drug. Ultrasound-guided injections directly visualize the needle in real-time as it pierces the skin to enter the target site. Physicians can perform ultrasound scanning immediately after injections to visualize the location of the corticosteroids which presents as echogenic lines or foci [28].

Our results are consistent with previous systematic reviews and meta-analyses [29, 30]; however, the present study differs from most of those studies because it considers multiple shoulder diseases, such as subacromial impingement syndrome, adhesive capsulitis, subacromial bursitis, biceps tendonitis were included, differences in mean changes between baseline and 6 weeks after injection were calculated, subgroup analyses according to the VAS score and functional score were performed, and a larger number of eligible studies (18 studies) were included in the meta-analysis.

A meta-analysis in 2013 by Sage et al., enrolling six papers with a total of 307 patients, examined US-guided and indicated a significant difference in shoulder pain and abduction in favour of US-guided compared to landmark steroid injection. However, no significant difference was found between the injection methods regarding shoulder function [29]. Soh et al. reviewed two RCTs including 101 patients with shoulder pain in 2011. Ultrasound-guided injections was related to significant improvement in shoulder pain and function at 6 weeks after injection [7]. Another meta-analysis in 2005 reported that subacromial injections of CS are effective for improvement for rotator cuff tendonitis up to 9-months. They are also probably more effective than NSAID medication [30].

Although the results may be correlated to the poorly controlled blinding of participants, it would be expected that the placebo effect to be minimal when both groups receive the same injection [16, 21]. Improvement in pain score may be justified due to the more patient comfort and less needle use with US-guided CS injections, despite not being investigated. Regarding the functional score improvement, there was significant heterogeneity between the included studies in addition to a small clinical effect [9, 16, 21]. Of note, most of the enrolled studies involved patients with chronic symptoms, which might have limited functional gain from CS injection alone, making it difficult to assess if there was a difference between groups.

The cost-effectiveness of US-guided injection of CS was lacking among the eligible studies. Although our results presented a superiority of US-guided over landmark-guided CS injections, the studies are small and did not evaluate cost-effectiveness. Similarly, a recent systematic review [31] investigating the value of imaging techniques to guide procedures in patients with rheumatic and musculoskeletal diseases found that data on cost-effectiveness is sparse. According to a consensus statement from the American Medical Society for Sports Medicine, there is preliminary evidence that US-guided injections are more cost-effective than landmark-guided injections [32] However, the cost-effectiveness of the US-guided method depends on the physician’s practice [33] In addition, there is evidence that providing training to physicians on the landmark application of CS injection is also cost-effective [34].

### Strengths and limitations

The current meta-analysis included up-to-data RCTs (up to 2021). We performed a comprehensive search using many electronic databases and we adhered to the PRISMA checklist when reporting this manuscript. We calculated the mean change difference, which provides a more accurate method to detect changes and considers any variation at baseline. Given the variable functional scores, a subgroup analysis was conducted according to the type of score. Nevertheless, several limitations exist. The included studies had a short follow-up period (average 6 weeks). While this provides some short-term indication of results, it does not allow any judgments to be made on the efficacy of US-guided steroid injection in the longer term. The risk of bias ranged from low to moderate in the included studies with lack of blinding in most included studies. However, successful blinding of participants in practice was difficult to achieve. Another limitation was the small sample size, which ranged from 28 to 100 patients. To enhance the evidence base, these limitations should be considered carefully while conducting further, well-performed RCTs.

### Implications for clinical practice

Over the last decade, US-guided injections have achieved widespread use, especially among non-radiologists, substituting the traditional anatomical approach [35]. The US-guided steroid injection was the main approach for patients with adhesive capsulitis because it offered the least expensive ($1280) and most efficient (0.4096 quality-adjusted life years) option compared to landmark and fluoroscopy-guided CS injection [33]. Further, our results confirm the superiority of US-guided injections over landmark-guided injections with the use of corticosteroids. Also, if orthobiological treatment does not cause systemic effects, then accuracy will be even more important.

## Conclusion

US-guided CS injections showed significant improvement in pain and functional scores but no difference in disability scores compared with landmark injections. While both compared groups were comparable in terms of disability scores and side effects. Further adequately powered and well-performed RCTs are recommended to confirm these results. Also, future research on the cost-effectiveness of US-guided CS methods is needed.

## Supplementary Information

Below is the link to the electronic supplementary material.

Supplementary file1 (DOCX 79 kb)
