# Supplementary material for: Systematic review and meta-analysis on the effectiveness of ultrasound-guided versus landmark corticosteroid injection in the treatment of shoulder pain: an update
Source: J Ultrasound. 2022 May 6;26(3):593–604. doi: 10.1007/s40477-022-00684-1 (PMC10468470; doi:10.1007/s40477-022-00684-1)
Supplement: Supplementary file 1 — Supplementary file1 (DOCX 79 kb) [file 40477_2022_684_MOESM1_ESM.docx]

**Supplementary file**

**Search strategies**

**PubMed search:**

(blind[tiab] OR landmark[tiab] OR anatomical[tiab] OR clinical exam[tiab] OR image-guided[tiab] OR ultrasound[tiab] OR fluoroscopy[tiab]) AND (steroid[MH] OR corticosteroid[tiab] OR glucocorticoid[tiab] OR sub-acromial[tiab] OR subacromial[tiab] OR triamcinolone[tiab] OR methylprednisolone[tiab] OR hydrocortisone[tiab] OR prednisolone[tiab] OR cortisone[tiab] OR dexamethasone[tiab] OR betamethasone[tiab) AND (frozen shoulder[MH] OR shoulder[tiab] OR adhesive capsulitis[tiab] OR shoulder pain[tiab] OR Shoulder Impingement Syndrome[tiab] OR rotator cuff OR Bursitis[tiab])

**Scopus search:**

(blind OR image-guided OR ultrasound) AND (steroid OR corticosteroid OR glucocorticoid OR prednisolone OR cortisone) AND (frozen shoulder OR adhesive capsulitis OR shoulder pain OR Shoulder Impingement Syndrome). All terms in all fields

**Web of science search: From 1900 to 2021**

(blind OR landmark OR anatomical OR clinical exam OR image-guided OR ultrasound OR fluoroscopy) AND (steroid OR corticosteroid OR glucocorticoid OR sub-acromial or subacromial OR triamcinolone OR methylprednisolone OR hydrocortisone OR prednisolone OR cortisone OR dexamethasone OR betamethasone) AND (frozen shoulder OR shoulder OR adhesive capsulitis OR shoulder pain OR Shoulder Impingement Syndrome OR rotator cuff OR Bursitis). all terms in title search.

**Cochrane CENTRAL search:**

(blind OR landmark OR anatomical OR clinical exam OR image-guided OR ultrasound OR fluoroscopy) AND (steroid OR corticosteroid OR glucocorticoid OR sub-acromial or subacromial OR triamcinolone OR methylprednisolone OR hydrocortisone OR prednisolone OR cortisone OR dexamethasone OR betamethasone) AND (frozen shoulder OR shoulder OR adhesive capsulitis OR shoulder pain OR Shoulder Impingement Syndrome OR rotator cuff OR Bursitis). All terms in title, abstract, keyword search

**EBSCO search: From 1958 to 2021**

(blind OR landmark OR anatomical OR clinical exam OR image-guided OR ultrasound OR fluoroscopy) AND (steroid OR corticosteroid OR glucocorticoid OR sub-acromial or subacromial OR triamcinolone OR methylprednisolone OR hydrocortisone OR prednisolone OR cortisone OR dexamethasone OR betamethasone) AND (frozen shoulder OR shoulder OR adhesive capsulitis OR shoulder pain OR Shoulder Impingement Syndrome OR rotator cuff OR Bursitis). All terms in title search.

**List of excluded studies**

| Author | Year | Reason of exclusion | Link |
| --- | --- | --- | --- |
| Kim et al | 2021 | Interventions are not of interest | https://pubmed.ncbi.nlm.nih.gov/34078012/ |
| Hsu et al | 2021 | Interventions are not of interest | https://pubmed.ncbi.nlm.nih.gov/33338463/ |
| Dumoulin et al | 2021 | Interventions are not of interest | https://pubmed.ncbi.nlm.nih.gov/33719606/ |
| Wang et al | 2019 | Interventions are not of interest | https://pubmed.ncbi.nlm.nih.gov/31150601/ |
| Farfaras et al | 2018 | Interventions are not of interest | https://pubmed.ncbi.nlm.nih.gov/29543510/ |
| Valtierra L et al | 2018 | Interventions are not of interest | https://pubmed.ncbi.nlm.nih.gov/29777953/ |
| Ekeberg et al | 2009 | Interventions are not of interest | https://pubmed.ncbi.nlm.nih.gov/19168537/ |
| Prestgaard et al | 2015 | Interventions are not of interest | https://pubmed.ncbi.nlm.nih.gov/19168537/ |
| Gyftopoulos et al | 2018 | Outcomes are not of interest | https://pubmed.ncbi.nlm.nih.gov/29629805/ |
| Malahias et al | 2018 | Population is not of interest | https://pubmed.ncbi.nlm.nih.gov/30404123/ |
| Nam et al | 2013 | Population is not of interest | https://pubmed.ncbi.nlm.nih.gov/23934387/ |
| Coene et al | 2017 | Thesis | https://open.bu.edu/handle/2144/23753 |

**Risk of bias assessment of included trials according to the Cochrane risk of bias tool**

| Trial | Random sequence generation | Allocation sequence concealment | Blinding of participants and personnel | Blinding of outcome assessment | Incomplete outcome data | Selective outcome reporting | Other potential sources of bias |
| --- | --- | --- | --- | --- | --- | --- | --- |
| Cho et al 2021 | Low | Low | Low | Low | Low | Low | Low |
| Akbari et al 2020 | Low | Low | Unclear | Unclear | Low | Low | Low |
| Yiannakopoulos et al 2020 | Low | Low | Unclear | Unclear | Low | Low | Low |
| Bhayana et al 2018 | Low | Low | Unclear | Unclear | Low | Low | Low |
| Raeissadat et al 2017 | Low | Low | Low | Unclear | Low | Low | Low |
| Cole et al 2015 | Low | Low | Low | Unclear | Low | Low | Low |
| Haghighat et al 2016 | Low | Low | Unclear | Unclear | Low | Low | Low |
| Saeed et al 2014 | Low | Low | Unclear | Low | High | Low | Low |
| Hsieh et al 2013 | Low | Low | High | Low | Low | Low | Low |
| Dogu et al 2012 | Low | Low | Low | Unclear | Low | Low | Low |
| Zufferey et al 2012 | Low | Low | High | Low | Low | Low | Low |
| Hashiuchi et al 2011 | Low | Low | Unclear | Unclear | Low | Low | Low |
| Zhang et al 2011 | Low | Low | Unclear | Unclear | Low | Low | Low |
| Panditaratne et al 2010 | High | High | High | High | Low | Low | Low |
| Lee et al 2009 | Low | Low | Unclear | Low | Low | Low | Low |
| Ucuncu et al 2009 | Low | Low | High | Low | Low | Low | Low |
| Chen et al 2006 | High | High | High | High | Low | High | Low |
| Naredo et al 2004 | Low | Low | Unclear | Low | Low | Low | Low |

**Leave-one-out-analysis**


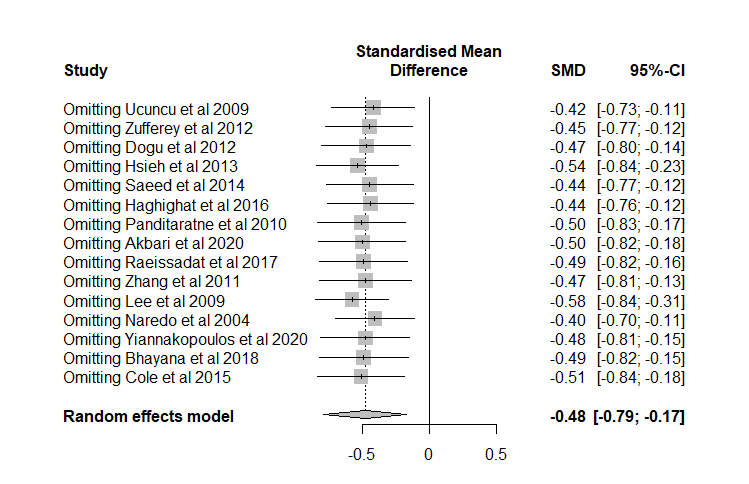


Results of leave-one-out method in sensitivity analysis in terms of visual analogue scale.


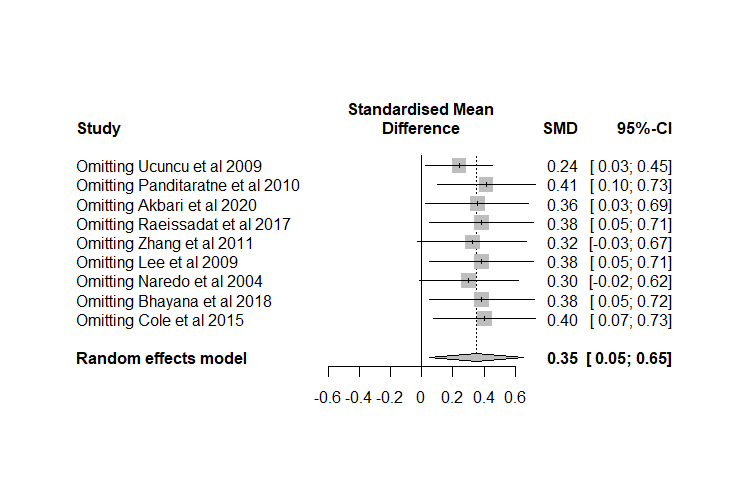


Results of leave-one-out method in sensitivity analysis in terms of shoulder function scores.


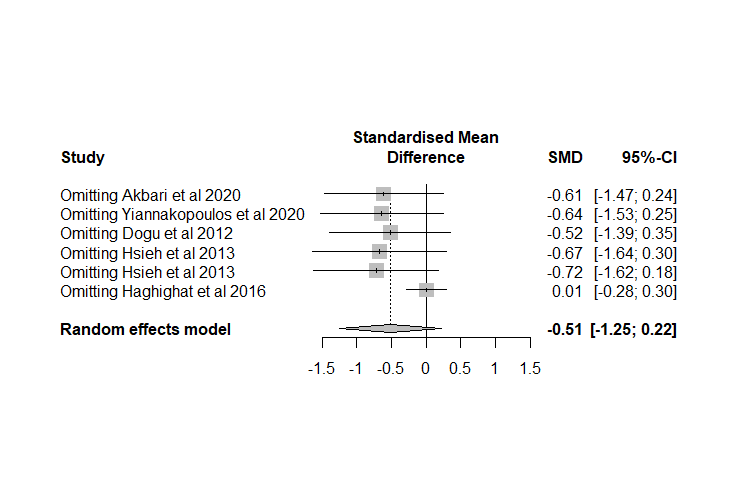


Results of leave one-out method in sensitivity analysis in terms of shoulder function scores.

**Funnel plots**


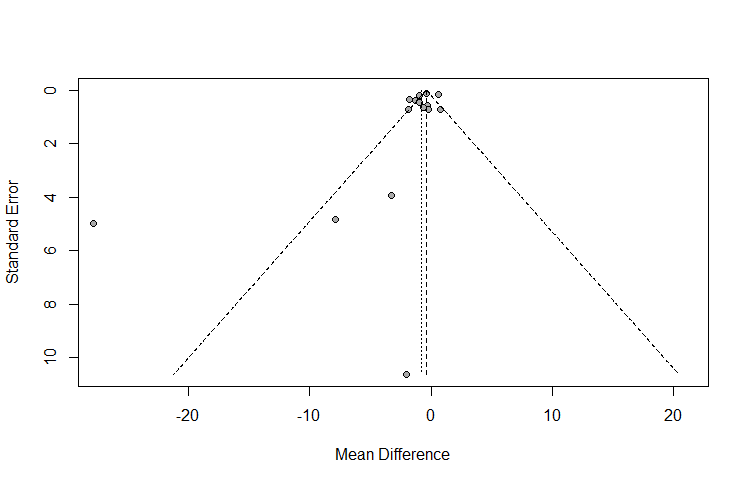


Funnel plot for studies of visual analogue scale. The dashed lines are pseudo 95% confidence limits. The x-axis represents the mean difference on a logarithmic scale, while the y-axis points to the standard error of the mean difference on a logarithmic scale.


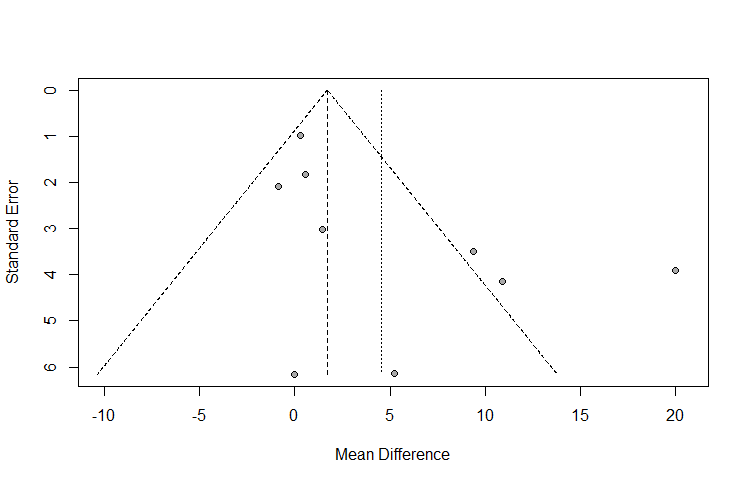


Funnel plot for studies on shoulder function scores. The dashed lines are pseudo 95% confidence limits. The x-axis represents the mean difference on a logarithmic scale, while the y-axis points to the standard error of the mean difference on a logarithmic scale.


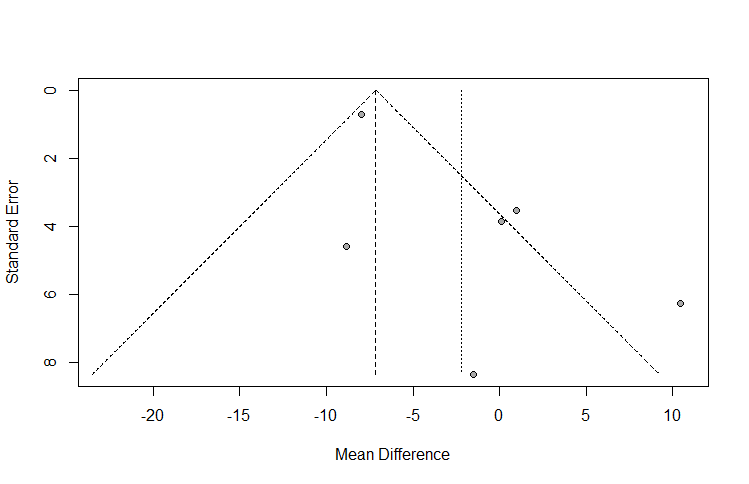


Funnel plot for studies on shoulder disabilities score. The dashed lines are pseudo 95% confidence limits. The x-axis represents the mean difference on a logarithmic scale, while the y-axis points to the standard error of the mean difference on a logarithmic scale.


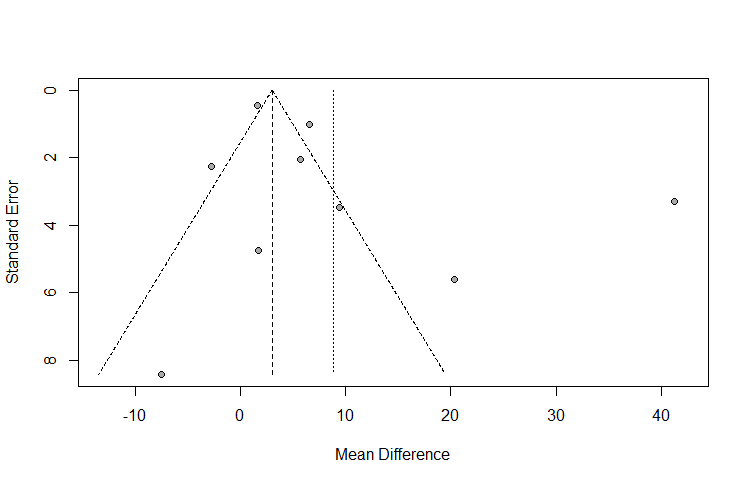


Funnel plot for studies on shoulder abduction degree. The dashed lines are pseudo 95% confidence limits. The x-axis represents the mean difference on a logarithmic scale, while the y-axis points to the standard error of the mean difference on a logarithmic scale.


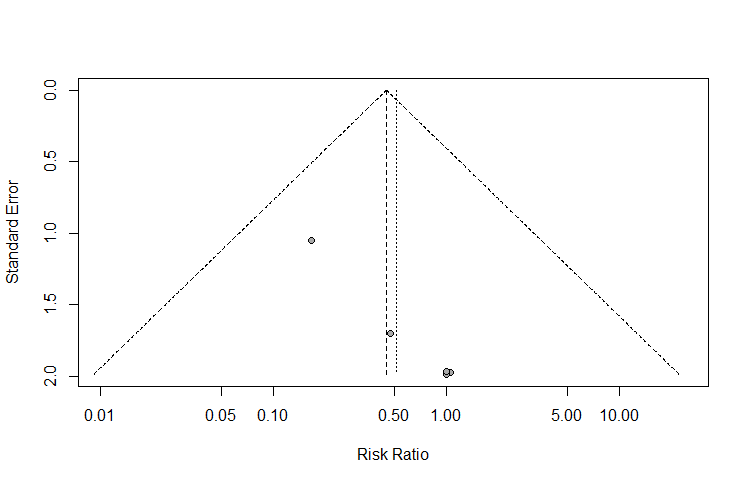


Funnel plot for studies on side effects. The dashed lines are pseudo 95% confidence limits. The x-axis represents the risk ratio on a logarithmic scale, while the y-axis points to the standard error of the risk ratio on a logarithmic scale.
